# A PKA/cdc42 Signaling Axis Restricts Angiogenic Sprouting by Regulating Podosome Rosette Biogenesis and Matrix Remodeling

**DOI:** 10.1038/s41598-018-37805-y

**Published:** 2019-02-20

**Authors:** J. L. MacKeil, P. Brzezinska, J. Burke-Kleinman, A. W. Craig, C. J. B. Nicol, D. H. Maurice

**Affiliations:** 1 Department of Biomedical and Molecular Sciences, Queen’s University, Kingston, Ontario K7L 3N6 Canada; 2 Department of Pathology and Molecular Medicine, Queen’s University, Kingston, Ontario K7L 3N6 Canada

## Abstract

Angiogenic sprouting can contribute adaptively, or mal-adaptively, to a myriad of conditions including ischemic heart disease and cancer. While the cellular and molecular systems that regulate tip *versus* stalk endothelial cell (EC) specification during angiogenesis are known, those systems that regulate their distinct actions remain poorly understood. Pre-clinical and clinical findings support sustained adrenergic signaling in promoting angiogenesis, but links between adrenergic signaling and angiogenesis are lacking; importantly, adrenergic agents alter the activation status of the cAMP signaling system. Here, we show that the cAMP effector, PKA, acts in a cell autonomous fashion to constitutively reduce the *in vitro* and *ex vivo* angiogenic sprouting capacity of ECs. At a cellular level, we observed that silencing or inhibiting PKA in human ECs increased their invasive capacity, their generation of podosome rosettes and, consequently, their ability to degrade a collagen matrix. While inhibition of either Src-family kinases or of cdc42 reduced these events in control ECs, only cdc42 inhibition, or silencing, significantly impacted them in PKA(Cα)-silenced ECs. Consistent with these findings, cell-based measurements of cdc42 activity revealed that PKA activation inhibits EC cdc42 activity, at least in part, by promoting its interaction with the inhibitory regulator, guanine nucleotide dissociation inhibitor-α (RhoGDIα).

## Introduction

Angiogenesis, the growth of blood vessels from pre-exiting vascular structures, is a critical developmental event *in utero* and in the revascularization of damaged or ischemic tissues in the adult^1^. In addition, angiogenesis contributes, either adaptively or mal-adaptively, to a myriad of conditions including ischemic heart disease and cancer^2–4^. Initiation of angiogenesis results when tissue hypoxia promotes a surge of the pro-angiogenic factor, vascular endothelial growth factor (VEGF). VEGF, via actions coordinated through its receptor, VEGFR2, promotes the conversion of quiescent endothelial cells (ECs) in local vascular structures to an activated “tip cell” phenotype^5–7^. Endothelial tip cells guide the growth of newly forming vessels and mediate contacts with existing vascular structures to form an anastomosing network^8–10^. Activation of tip cells through VEGF/VEGFR2 also upregulates expression of the Notch ligand, delta-like ligand 4 (Dll4)^11–14^; Dll4, in turn, binds Notch1 receptors on neighboring cells to initiate Notch signaling and induce a stalk EC phenotype. In contrast to tip ECs, stalk ECs migrate and proliferate to promote lengthening and maturation of the newly developing vessel.

Extensive research has allowed for identification of the signaling pathways that coordinate tip and stalk specification during angiogenesis. In contrast, our understanding of the systems that regulate the pro-angiogenic functions of these two phenotypically distinct ECs remains in its infancy. For instance, there is limited understanding of the systems that coordinate the ability of tip ECs to establish VEGF stimulated polarity, extend cellular projections toward the VEGF gradient, degrade extracellular matrix (ECM) and migrate during angiogenic sprouting. In these latter events, matrix degradation is catalyzed by local recruitment and activation of membrane type-1 metalloproteinases (e.g. MMP14), together with MMP14-activated MMPs (e.g. MMP2 and MMP9), all of which serve as critical steps for subsequent tip EC matrix invasion^15,16^. Tip EC MMP14 localization occurs predominantly at cellular regions enriched in podosomes, adhesive actin-based structures that demark sites of ECM remodeling in invasive cells^17,18^. Although numerous separate signaling systems coordinate podosome formation in cells, including ECs, their relative contributions during angiogenic sprouting are unclear^19^. For instance, while compelling evidence supports involvement of Src family kinases, Rho family GTPases (i.e. cell division control protein 42 homolog, cdc42) and phosphoinositide 3-kinases as key regulators of podosome biogenesis in ECs^20^, their relative dominance during distinct invasive contexts remains unknown. Moreover, individual EC podosomes can be used to form larger (5–10 µm diameter) actin-based, ECM degrading, cellular superstructures; these superstructures are referred to as podosome “rosettes”. Currently, whether and how these signaling systems control the organization of podosomes into higher order podosome rosettes is virtually unknown.

Cyclic 3′,5′-adenosine monophosphate (cAMP) was the first intracellular molecule shown to act as a second messenger, allowing cells to faithfully respond to signals encoded by primary extracellular messengers. Since its discovery, numerous physiological agents have been shown to regulate cellular functions through actions mediated largely by the cAMP-effectors, Protein Kinase A (PKA), Exchange Protein Activated by cAMP (EPAC) and cyclic nucleotide-gated ion channels. In ECs, the cAMP system decodes and integrates signaling from numerous primary messengers including hormones, transmitters and the mechanical forces exerted by fluid shear stress. Although the ubiquity with which cAMP-signaling acts in cells makes this system an attractive therapeutic target, it also limits the specificity of many of the drugs developed for this purpose. Indeed, although cAMP was first described over 60 years ago^21^, only relatively recent findings have highlighted how this ubiquitous second messenger simultaneously regulates countless events in virtually all types of mammalian cells^22^. Presently, a consensus exists that specificity of cAMP signaling is achieved when its effectors (PKA, EPAC or cyclic nucleotide-gated ion channels), act within intracellular signaling compartments, not globally throughout the cell^23–25^. Moreover, it is now clear that these signaling compartments form when individual components of the cAMP signaling system, including adenylyl cyclases, cAMP effectors and cAMP-hydrolyzing phosphodiesterases, generate macromolecular complexes (termed “cAMP-signalosomes”) within numerous distinct subcellular domains. Consistent with this model, we, and others, have shown that regulating selective cAMP-sensitive cellular functions is possible by targeting actions of a selected individual cAMP-effector within an individual cAMP signalosome^23–25^. Indeed, when tested, this approach has yielded greater selectivity than was achieved by targeting cAMP effectors globally throughout the cell.

To date, studies assessing the role of cAMP signaling in EC sprouting angiogenesis are few and contradictory. While several studies report that increases in cAMP correlate with inhibition of EC angiogenesis^26–28^, others indicate that activation of either PKA or EPAC, the dominant EC cAMP effectors, promote certain pro-angiogenic events^29,30^. To comprehensively address the roles of PKA or EPAC in sprouting angiogenesis, we used several model systems, including a mouse retinal model of sprouting angiogenesis, human aterial EC (HAEC) and microvascular EC (HMVEC) cultures. Overall, our findings highlight a critical inhibitory role for PKA, but not EPAC, in sprouting angiogenesis. Specifically, we report that knocking down levels of either of the PKA catalytic domains (PKACα or PKACβ), or selectively inhibiting PKA activity, promotes EC invasion, an effect which markedly increases sprouting angiogenesis. Conversely, PKA activation impaired EC invasion and sprouting. At a cellular level, we demonstrate that PKA regulates EC angiogenic sprouting through two interdependent cellular events. Thus, while PKA modestly antagonized VEGF-induced podosome biogenesis in ECs, it markedly inhibited formation of podosomal superstructures (i.e. podosome “rosettes”) in these cells, which in turn tightly regulates matrix degradation. At a molecular level, while our studies confirm a role for both Src family kinases and cdc42 in podosome rosette biogenesis and in EC sprouting, they unmask a critical role for PKA regulation of cdc42 during these events. Furthermore, we provide evidence that PKA regulates cdc42 activity by dynamically regulating its association with an inhibitory regulator, guanine nucleotide dissociation inhibitor α (RhoGDIα). Taken together, our data provide insight into a novel PKA-RhoGDI-cdc42 signaling axis and highlight how controlling PKA expression, or its activity, in ECs during angiogenesis may represent a novel therapeutic strategy to control sprouting angiogenesis in humans.

## Results

### Endothelial PKA, but not EPAC, negatively regulates VEGF-induced angiogenic sprouting

To explore the functional role of the cAMP effectors expressed in human ECs (PKA and EPAC) in angiogenic sprouting, we utilized a “mosaic spheroid” competitive sprouting angiogenesis assay^31^. In this assay, ECs with one of the cAMP-effectors selectively knocked down competed against control ECs transfected with a non-targeting siRNA for the “tip position” in newly forming angiogenic sprouts. Dominance in tip cell occupancy by one of these cells reflects its superior capacity to generate angiogenic sprouts. In these studies, we used both human arterial (HA) and human microvascular (HMV) ECs. Selective silencing of the individual PKA catalytic subunits (Cα or Cβ) expressed in these ECs was achieved using several distinct siRNAs and the effectiveness of this strategy was confirmed at the level of mRNA (qPCR) (Supplementary Fig. S1a), protein (immunoblotting) (Supplementary Fig. S1b) and PKA activity (Supplementary Fig. S1c). Effectiveness of silencing EPAC1, the sole EPAC expressed in human ECs, by this strategy was similarly confirmed at the mRNA level (Supplementary Fig. S1a) and anti-EPAC1 immunoblotting (Supplementary Fig. S1b). To readily identify cells occupying the tip position within individual sprouts, we labelled control ECs with CellTracker™ Red and cAMP effector-targeted ECs with CellTracker™ Green.

**Figure 1:**
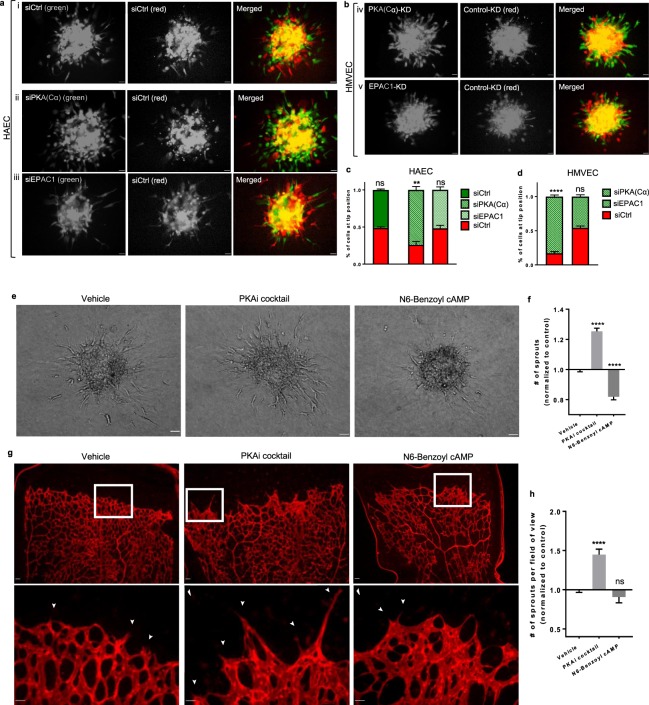
PKA coordinates the intrinsic angiogenic sprouting potential of human ECs *in vitro* and murine ECs *ex vivo.* **(a,b)** Representative images depicting results of competitive sprouting angiogenesis in mosaic spheroids containing 1:1 mixtures of either (i) control-control, (ii, iv) control-PKA(Cα)-KD, or (iii, v) control-EPAC1-KD **(a)** HAECs or **(b)** HMVECs. EC identity in individual spheroids was determined through differentially labelling with either CellTracker™ Green, or CellTracker™ Red, respectively. Scale bars denote 50 μm. **(c,d)** The competitive sprouting was assessed by quantifying tip cell occupancy of CellTracker™ Green- or CellTracker™ Red-labelled cells in individual (**c**) HAEC or (**d**) HMVEC spheroids. Values are expressed as a percentage of the total number of sprouts formed in each spheroid (n = 3; **p<0.01, ****p<0.0001 in Student’s unpaired t-test). **(e)** Representative images of sprouting in HAEC spheroids treated with either vehicle, a PKAi cocktail or N6-Benzoyl-cAMP; scale bars, 50 μm. **(f)** Quantification of HAEC sprouting; values are normalized to the vehicle treated control (n = 3; ****p<0.0001 in Kruskal-Wallis test). (**g**) Representative low and high magnification images of the sprouting front of an *ex vivo* mouse retinal model of angiogenesis. Retinas were isolated from postnatal day 5 mice and treated with the PKAi cocktail (n = 9), N6-Benzoyl-cAMP (n = 6), or the vehicle (n=13) for 8 hours. The retinal vasculature was visualized with IsolectinB4; angiogenic activity was evaluated by EC sprouting at the vascular front (➤). Scale bars in top panel denote 50 µm; scale bars in lower panel denote 25 µm. **(h)** Quantification of the number of sprouts emerging from the front of the vascular plexus per field of view; values are normalized to the control (vehicle) ****p<0.0001 in one-way ANOVA.

While neither CellTracker™ Green or CellTracker™ Red labelled HAEC transfected with the control siRNA showed a preference for tip cell occupancy (Fig. 1a(i)), HAEC and HMVEC mosaic spheroids containing equal numbers of control- and PKA(Cα)-knockdown (KD) cells yielded sprouts in which PKA(Cα)-silenced cells predominantly occupied the tip position (≥75%) (Fig. 1a(ii),b(i),c,d). Since this hyper-sprouting effect was observed in cells transfected with either of two distinct PKA(Cα)-targeting siRNAs (Supplementary Fig. S1d), as well as in ECs transfected with a PKA(Cβ)-targeting siRNA (Supplementary Fig. S1e), we conclude that the increased capacity of the PKA(C)-targeted cells to form sprouts is related to a reduction in total cellular PKA(C). Interestingly, angiogenic sprouts from HAEC, or HMVEC, mosaic spheroid containing an equal proportion of control- and EPAC1-knockdown cells showed no preference for tip cell occupancy (Fig. 1a(iii),b(ii),c,d). Given the high efficiency of EPAC1 knockdown (KD) (Supplementary Fig. S1a,b), and our previous work showing that EPAC1-KD HAECs are less responsive to changes in fluid shear stresses^32^, we conclude that PKA(C), but not EPAC1, critically regulates the angiogenic sprouting capacity of both human arterial or microvascular ECs.

Consistent with the idea that reducing levels of PKA(C) unmasked an inhibitory influence of this kinase on the intrinsic sprouting capacity of both HAECs and HMVECs, altering PKA activity pharmacologically also influenced their sprouting capacity. Thus, while inhibition of PKA^33,34^ in HAEC spheroids promoted sprouting, addition of a selective PKA-activating cAMP-analogue, N6-Benzoyl-cAMP, markedly inhibited such sprouting (Fig. 1e,f). These effects directly correlated with the impact of these treatments on PKA activity, assessed by the phosphorylation status of a *bona fide* PKA substrate in these cells (Supplementary Fig. S1f). While silencing EPAC1 did not alter sprouting in either HAECs, or HMVECs (Fig. 1a(iii),b(ii),c,d), addition of a selective EPAC inhibitor, CE3F4^35^, did reduced HAEC sprouting by ~20% in both control- and PKA(C)-KD HAECs (Supplementary Fig. S1g). However, since CE3F4 did not preferentially impact sprouting in PKA(Cα)-KD ECs, and an EPAC-selective agonist, 8-CPT-2’-O-Me-cAMP, did not alter sprouting in either control- or PKA(C)-KD spheroids, our findings are inconsistent with EPAC1 having a significant role in EC sprouting or in the hyper-sprouting observed in ECs in which PKA was inhibited or silenced.

Tip cell specification in response to VEGF requires enhanced expression of VEGFR2 and Dll4^11–14^. Given that increased levels of VEGFR2 and/or Dll4 might impart a substantial advantage in angiogenic sprouting, we next investigated whether levels of these tip cell markers were elevated in PKA(C)-KD HAECs, given their enhanced sprouting capacity. Our findings are inconsistent with the hypothesis that PKA(C)-KD HAECs are “primed” to adopt a tip EC phenotype based on their expression of these tip cell-enriched genes (Supplementary Fig. S1h–j). Indeed, levels of VEGFR2 were similar in control-KD and PKA(C)-KD HAECs (Supplementary Fig. S1h, i), while expression of Dll4 mRNA was significantly reduced in PKA(C)-KD HAECs when compared to control-KD cells (Supplementary Fig. S1j). Thus, PKA(C)-KD HAECs are not imparted with a competitive advantage during angiogenesis by upregulating genes associated with tip cell sprouting. Interestingly, these findings are consistent with previous work^36^ in which endothelial-specific expression of a dominant-negative PKA caused vascular hyper-sprouting in the mouse retina, independently of Notch-signaling.

Since our studies in HAECs support the premise that PKA activity critically regulates angiogenic sprouting, we next investigated the role of PKA in the more physiologically relevant *ex vivo* mouse retinal model of angiogenesis^37,38^. Pharmacologic PKA inhibition in this model significantly increases the number of EC sprouts emerging at the vascular front of the developing retina, a sign of retinal vascular hyper-sprouting (Fig. 1g,h). Consistent with the idea that endogenous PKA activity is sufficient to control retinal vascular sprouting in this developmental model, addition of the PKA-selective activator (N6-Benzoyl-cAMP), which markedly inhibited sprouting by HAEC spheroids, only modestly decreased sprouting in the isolated mouse retina (Fig. 1g,h). Overall, these data support a critical role for PKA activity in regulating basal human sprouting angiogenesis and in the in the mouse retina.

### PKA negatively regulates the invasive potential of HAECs

**Figure 2:**
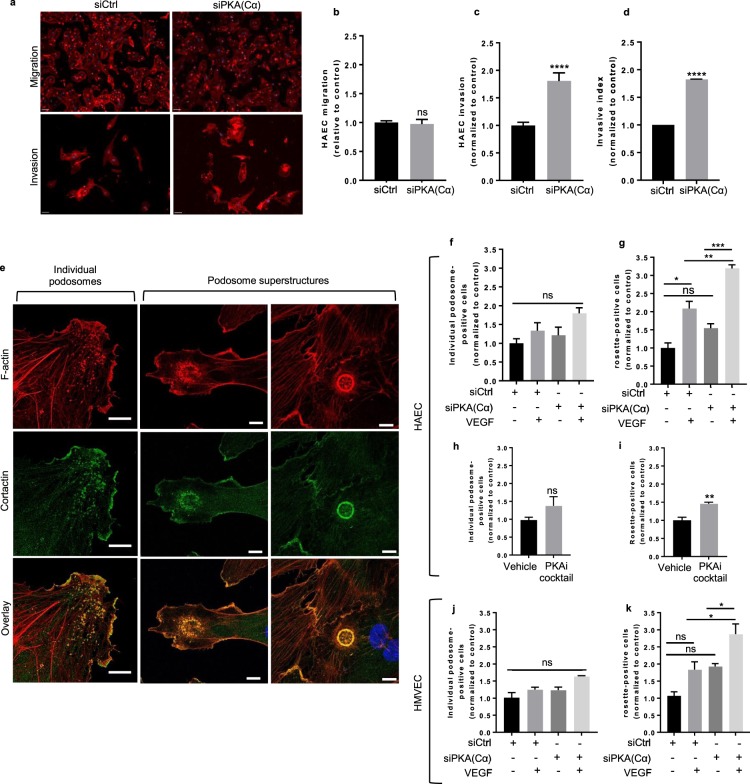
PKA(Cα)-silencing promotes invasion and enhances the formation of podosome superstructures in ECs. **(a)** Representative images of VEGF-induced chemotaxis and chemoinvasion. Actin and nuclei in these cells were visualized by staining with TRITC-conjugated phalloidin and with DAPI, respectively; scale bars, 50 μm. **(b–d)** Quantification of whole cell **(b)** migration (n = 3), **(c)** whole cell invasion (n = 3), and **(d)** the invasive index of control- and PKA(Cα)-knockdown HAECs. Invasive index was calculated as the ratio of invaded:migrated cells. All values are normalized to the control knockdown; ****p<0.0001 using Mann-Whitney U test. **(e)** Representative high-resolution confocal images of individual podosomes and podosome superstructures (ie rosettes) in ECs. Podosomes or podosome superstructures were identified based on the co-localization of actin and cortactin staining; scale bars, 10 μm. Quantification of the percent of HAECs and/or HMVECs with **(f,j)** individual podosomes or **(g,k)** posodosome rosettes in control- and PKA(Cα)-knockdown cells cultured under basal conditions and following 24hr VEGF stimulation (25 ng/ml). (N = 3; *p<0.05, **p<0.01, ***p<0.001 using one-way ANOVA). **(h,i)** Quantification of the percent of HAECs with **(h)** individual podosome or **(i)** podosome rosettes following 24 h hour treatment with the PKAi cocktail or vehicle in the presence of VEGF (25 ng/ml). Values are normalized to the vehicle-treated control (n = 3; **p<0.01 using a Student’s unpaired t-test).

Given that silencing the catalytic subunits of PKA, or pharmacological inhibition of PKA activity, increased angiogenic sprouting, while PKA activation impaired sprouting, we next sought to identify the individual cellular event(s) critical to angiogenic sprouting that were sensitive to these changes in PKA. To this end, we modified an established protocol^39^ to differentiate the effects of PKA on the invasive and migratory potentials of these cells. Interestingly, the ability of control or PKA(Cα)-KD HAECs to migrate in response to a VEGF gradient were indistinguishable (Fig. 2a,b). In contrast, when these cells were required to invade a collagen gel in response to a VEGF gradient, PKA(Cα)-KD HAECs were markedly more efficient than control cells (Fig. 2a,c). Thus, PKA(Cα)-KD HAECs exhibited a markedly elevated invasive capacity (invasive index) relative to control HAECs (Fig. 2d). Taken together, these data signify that PKA negatively regulates the intrinsic invasive capacity of HAECs and suggest that PKA activity in HAECs acts to reduce the angiogenic sprouting capacity of these cells by limiting their ability to invade the surrounding ECM.

### PKA regulates podosome rosette formation

A large body of evidence supports the premise that matrix remodeling by ECs occurs predominantly at sites enriched in podosomes^40,41^. As such, we investigated whether altering PKA(Cα) levels influenced the ability of HAECs, or HMVECs, to form these small (0.5–1 µm diameter) actin-rich invasive structures (Fig. 2e). Under basal conditions, we observed no difference in the number of control or PKA(Cα)-KD HAECs (Fig. 2f), or HMVECs (Fig. 2j), that produced podosome. Indeed, while addition of VEGF increased the fraction of these cells that generated podosomes, the impact of this angiogenic stimulus on podosome biogenesis was not materially different between control-KD and PKA(Cα)-KD cells. Similarly, PKA inhibition did not significantly alter the number of HAECs that generated individual podosomes (Fig. 2h).

Although individual EC podosomes coordinate hyper-localized ECM degradation, these sub-micron structures can also be organized into larger (5–10 µm in diameter) cellular superstructures (ie podosome rosettes) (Fig. 2e). While the systems that control the formation of rosettes from individual podosomes are largely unknown, it is clear that these structures are required for vascular sprouting^18,42^. Given that PKA(Cα)-KD ECs exhibited a hyper-sprouting phenotype compared control cells, we reasoned that cells with reduced PKA(Cα) levels might have an enhanced capacity to generate podosome rosettes. In support of this hypothesis, the percentage of HAECs or HMVECs expressing podosome rosettes was higher in PKA(Cα)-KD compared to control cells, both under basal conditions and in the presence of added VEGF (Fig. 2g,k). Furthermore, selective inhibition of PKA markedly promoted podosome rosette formation in these cells (Fig. 2i). Overall, these data highlight a crucial role for PKA(C) in the regulated formation of podosome rosettes in both HAECs and HMVECs, and are consistent the effects of PKA(C)-KD on EC invasion and sprouting.

### PKA regulates podosome rosette-mediated, MMP-dependent, collagen matrix degradation

**Figure 3:**
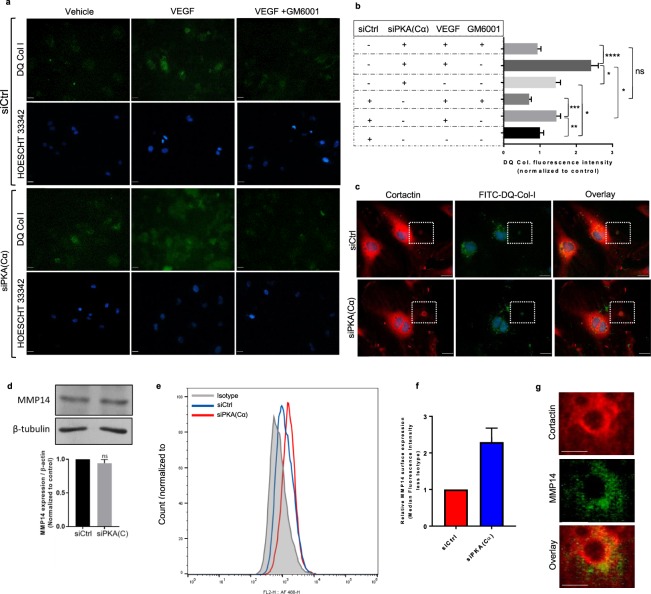
PKA(Cα)-silencing enhances MT1-MMP surface expression and MMP-dependent matrix degradation. (**a**) Representative images showing DQ-Col-I degradation (green) by HOESCHT33342-labelled HAECs which had been transfected previously with either a control siRNA (top 2 rows) or a PKA(Cα) siRNA (bottom 2 rows). DQ-Col-1 degradation was measured under basal conditions, in the presence of VEGF (25 ng/ml), or in the presence of VEGF and the broad spectrum MMP inhibitor GM6001 (10 μmol/L). **(b)** Quantification of DQ-Col-I degradation measured as the average FITC fluorescence intensity per cell. Values are normalized to vehicle treated control siRNA HAECs (n = 3; *p<0.05, **p<0.01, *** p<0.001, ****p<0.0001 by Kruskal-Wallis test). **(c)** Representative images of cortactin-stained HAECs following DQ-Col-I degradation. Matrix degradation, indicated by FITC fluorescent signal (green), is found within the core of podosome rosettes in control and PKA(Cα)-knockdown HAECS; scale bars, 50 μm. **(d)** Immunoblot analysis in control and PKA(Cα)-silenced indicates that PKA(Cα)-silencing does not significantly alter HAEC expression of MMP14 (n = 3). **(e)** Cell surface expression of MMP14 assessed by flow cytometry in non-permeabilized control and PKA(Cα)-KD HAECs using an AlexaFluor 488-conjugated anti-MMP14 antibody or mouse IgG2B Alexa Fluor® 488-conjugated isotype control. **(f)** Cell surface levels of MMP14 in PKA(Cα)-KD HAECs relative to controls (n = 2). **(g)** Representative images showing cell surface MMP14 and intracellular cortactin co-localization at a podosome rosette in HAECs.

To determine whether the hyper-sprouting phenotype observed in PKA(C)-KD human ECs correlated with their ability to degrade the extracellular matrix, we next assessed this capacity in control and PKA(Cα)-KD ECs. For these studies, collagen degradation was evaluated by measuring the fluorescence emitted from a fluorogenic collagen (i.e. DQ™ collagen) upon its hydrolysis by live ECs. As predicted, MMP-mediated (i.e. GM6001-sensitive) collagen degradation by control-KD HAECs under basal conditions was modest, but significantly increased upon the addition of VEGF (Fig. 3a,b). Consistent with results from our podosome rosette studies, collagen hydrolysis by PKA(Cα)-KD HAECs was significantly higher than that in control-KD ECs, both under basal conditions and in the presence of VEGF (Fig. 3a,b). To confirm the functionality of podosome rosettes, and correlate matrix degradation with rosette formation, we stained control or PKA(Cα)-KD HAECs for podosome markers (i.e. cortactin) following their incubation on a DQ-Col-I supplemented collagen-1 matrix. In these studies, we readily detected FITC-DQ collagen degradation products within the central core of cortactin-stained circular structures (i.e. rosettes) in both control and PKA(Cα)-KD HAECs (Fig. 3c). Overall, these data support the notion that increased formation of podosome rosettes in PKA(Cα)-KD ECs allows for more efficient matrix degradation and subsequent sprout formation, both under basal conditions and in the presence of added VEGF.

Since MMP14-mediated matrix remodeling is critical for EC sprouting, and for the activation of non-membrane bound MMPs, we considered the possibility that increased MMP14 expression in PKA(Cα)-KD HAECs might contribute to their hyper-sprouting capacity. In contrast to this hypothesis, immunoblot analysis revealed that total cellular MMP14 levels were equivalent between control- and PKA(Cα)-KD HAECs (Fig. 3d). Given that MMP14 is actively recruited to EC cell surface sites enriched in podosomes and podosome rosettes^43,44^, we next sought to determine whether cell surface levels of MMP14 were elevated in PKA(Cα)-KD human ECs compared to controls. Flow cytometry analysis revealed that cell surface expression of MMP14 in PKA(Cα)-KD HAECs was substantially elevated when compared to levels observed in control HAECs (Fig. 3e,f); immunostaining confirmed MMP14 recruitment to podosome rosettes (Fig. 3g). Thus, while levels of MMP14 were conserved between control- and PKA(Cα)-KD HAECs, greater surface exposure of the membrane-bound MMP in PKA(Cα)-KD HAECs corresponds with both increased numbers of podosome rosettes and enhanced matrix degradation in these cells.

### PKA regulates angiogenic sprouting and podosome rosette formation through cdc42

Overall, our data identify an intrinsic regulatory role for PKA in podosome rosette formation, MMP-mediated collagen degradation, cell invasion and ultimately, angiogenic sprouting. To begin to identify the mechanisms through which PKA might regulate these processes, we compared the activation status of several signaling systems known to regulate EC sprouting angiogenesis. First, to investigate whether knocking down HAEC PKA(Cα) simply sensitized these cells to actions of VEGF, we characterized the activation of the Akt, Erk1/2 and VEGFR2 signaling system, all of which have been reported to positively correlate with VEGF/VEGFR2-induced EC angiogenic sprouting^45–47^. Interestingly, while basal levels of Akt, Erk1/2 and VEGFR2 activation were modestly elevated in PKA(Cα)-KD HAECs compared to the control-KD cells, their activation upon addition of VEGF was equivalent (Supplementary Fig. S2). Thus, while PKA modestly regulates the basal level of activation of these systems, it does not materially alter their activation status in response to VEGF stimulation.

**Figure 4:**
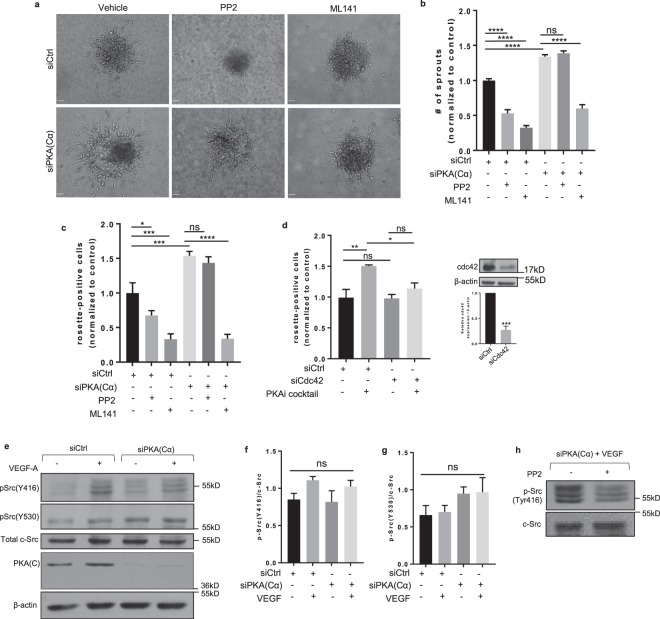
Enhanced EC sprouting and podosome rosette formation following PKA(Cα)-knockdown are largely mediated through cdc42. **(a)** Representative images of angiogenic sprouting in control or PKA(Cα)-silenced HAEC spheroids in the presence of vehicle, the src inhibitor (PP2, 10 μmol/L) or the cdc42 inhibitor (ML141, 10 μmol/L); scale bars denote 50 µm. **(b)** Quantification of EC sprouting in the spheroid angiogenesis model (n = 3; ****p<0.0001 by Kruskal-Wallis test). **(c)** The impact of src inhibition (PP2, 10 μmol/L) and cdc42 inhibition (ML141, 10 μmol/L) on the percent of control and PKA(Cα)-KD HAECs expressing podosome rosettes. **(d)** Quantification of the percent of cells expressing posodosome rosettes in control- and cdc42-KD HAECs. For podosome studies, cells were treated with the PKAi cocktail or the vehicle for 24 hours in the presence of VEGF (25 ng/ml). **(c,d)** N = 3; *p<0.05, **p<0.01, ***p<0.001, ****p<0.0001 by one-way ANOVA. **(e)** Western blot analysis of cdc42 knockdown efficiency (n = 3; ***p<0.001 in Student’s unpaired t-test). **(f)** Representative blot showing phosphorylation of the Src activation site (Tyr416) and inhibition site (Tyr530) in control and PKA(Cα)-knockdown HAECs under basal conditions and following VEGF stimulation (25 ng/mL). **(g,h)** Western blot analysis of Src phosphorylation at the **(g)** activation (Tyr416) and **(h)** inhibition (Tyr530) sites (n = 3). **(i)** Validation of Src inhibition by PP2 in PKA(C)-knockdown HAECs (representative Western blot; n = 2).

In addition to the Akt- and MAPK-signaling systems described above, numerous studies have invoked critical roles for Src family kinases, most commonly c-src itself, in EC angiogenic sprouting and in podosome biogenesis^20,48^. Since PKA activation was previously shown to activate the c-Src inhibitory kinase, c-Src kinase (Csk)^49^, in human umbilical vein-derived ECs, we hypothesized that PKA might regulate HAEC angiogenic sprouting through c-Src. To this end, we compared the impact of Src inhibition with its validated inhibitor, PP2, on sprouting angiogenesis in both control and PKA(Cα)-KD HAEC spheroids. As predicted, PP2 reduced HAEC sprouting in control-KD spheroids (Fig. 4a,b); however, the Src inhibitor was without effect on VEGF-induced sprouting angiogenesis in PKA(Cα)-KD spheroids (Fig. 4a,b). Given that enhanced sprouting in PKA(Cα)-KD HAECs and HMVECs was proportional to the increased podosome rosette formation in these cells, we next assessed the influence of PP2 on VEGF-induced podosome rosette formation in control and PKA(Cα)-KD HAECs. Consistent with results from spheroid angiogenesis assays, PP2 markedly reduced the fraction of control-KD ECs expressing podosome rosettes but did not impact podosome rosette formation in cells in which PKA(Cα) had been silenced (Fig. 4c). To determine if our findings were related to alterations in c-Src levels, or in the ability of PP2 to inhibit c-Src in PKA(C)-KD cells, we evaluated whether Src expression, activation by VEGF, or its inhibition by PP2 were altered in PKA(Cα)-KD HAECs. Results from these experiments confirmed that silencing EC PKA(C) did not alter c-Src levels, its activity or its sensitivity to PP2 in human ECs (Fig. 4e–h). Taken together, these data refute the notion that PKA(Cα)-KD promotes increased podosome rosette formation and angiogenic sprouting in HAECs largely by interfering with c-Src mediated pro-angiogenic events.

In addition to c-Src, the Rho-family GTPase, cdc42, has been reported to promote EC sprouting angiogenesis by stimulating podosome biogenesis^20,50^. Given that our studies were largely discordant with a role for Src in PKA-mediated regulation of HAEC sprouting and podosome rosette biogenesis, we next investigated the role of cdc42 in these PKA-dependent events. Remarkably, our data largely support a critical role for cdc42 in regulating the pro-angiogenic phenotype in PKA(Cα)-KD human ECs. Thus, unlike PP2, cdc42 inhibition with a cdc42-selective concentration of ML141 markedly reduced angiogenic sprouting (Fig. 4a,b) and podosome rosette formation (Fig. 4c) in both control and PKA(Cα)-KD HAECs. Furthermore, selective silencing of cdc42 in these cells abolished the ability of PKA inhibition to promote podosome rosette formation (Fig. 4d). Taken together, these data support a critical role for a PKA-cdc42 signaling axis in the regulation of sprouting angiogenesis.

### PKA-catalyzed RhoGDIα phosphorylation, cdc42-RhoGDIα interactions and cdc42 inhibition

**Figure 5:**
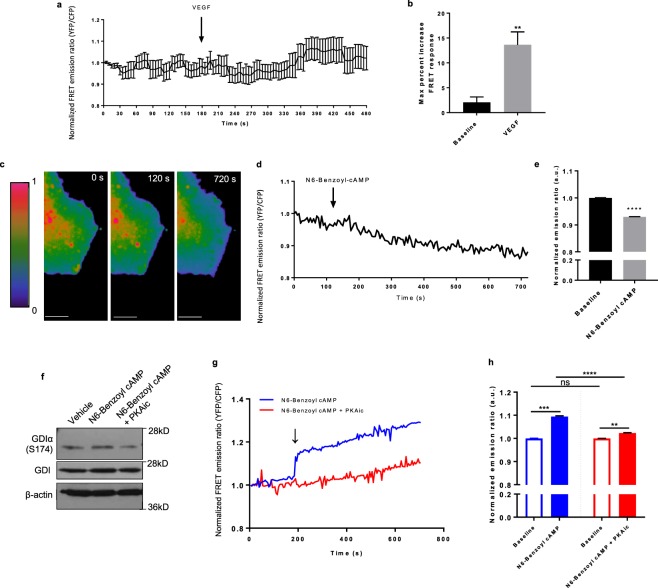
PKA regulates the association of cdc42 with its negative regulator, GDIα. **(a)** Plot of the average normalized FRET emission ratio (YFP/CFP) at baseline and following VEGF stimulation (25 ng/ml) in HAECs expressing the pTriEx4-Cdc42-2G sensor (n = 8 cells). **(b)** The maximum percent increase in the FRET response was calculated for the baseline (3min) and following VEGF (25 ng/ml) stimulation (5 min). **(c)** Representative heat maps of cdc42 activity in a HAEC expressing the pTriEx4-Cdc42-2G FRET biosensor. Panels show cdc42 activity at baseline (0s), at the time of 30 µmol/L N6-Benzoyl-cAMP addition (120 s), and following a 10 min incubation with the PKA activator (720 s); warmer colors represent high cdc42 activity, while cooler colors indicate low cdc42 activity. Scale bar, 20 µm. **(d)** Representative single-cell trace of the normalized FRET ratio response (CFP/YFP) at baseline and following the addition of N6-Benzoyl-cAMP (30 µmol/L) in a HAEC expressing the pTriEx4-Cdc42-2G sensor. **(e)** Quantification of the average FRET emission ratio at baseline and following N6-benzoyl cAMP addition (n = 13 cells; ****p<0.0001 in Student’s unpaired t-test). **(f)** Representative blot of GDIα phosphorylation at S174 in cells treated with N6-Benzoyl cAMP (30 µmol/L), N6-Benzoyl and the PKAi cocktail, or the vehicle (n = 2). **(g)** Representative traces of the normalized FRET ratio response (YFP/CFP) at baseline and following stimulation with N6-Benzoyl-cAMP (30 µmol/L) or N6-Benzoyl-cAMP + PKAi cocktail in HAECs expressing the pTriEx-Antenna- GDI cdc42 sensor. **(h)** Quantification of the average FRET emission ratio at baseline, and following the addition of N6-Benzoyl-cAMP (n = 12 cells) or N6-Benzoyl-cAMP + PKAic (n = 16 cells); **p<0.01, ***p<0.001, ****p<0.0001 in Student’s unpaired t-test.

To directly investigate the manner through which PKA influenced cdc42 activity, and by extension EC sprouting and podosome rosette biogenesis, we made use of two distinct fluorescence resonance energy transfer (FRET)-based cdc42 biosensors. First, we utilized a cdc42-2G sensor^51^, which detects changes in cdc42 activity upon cdc42-GTP loading to the WASP N-terminal cdc42/Rac1interactive binding motif (CRIB) domain. Validating this approach, addition of VEGF (25 ng/ml) increased cdc42 activity as measured by an increase in the FRET emission ratio (Fig. 5a,b). Consistent with the idea that PKA regulates HAEC sprouting by inhibiting cdc42 activation, addition of the selective PKA activating cAMP analogue, N6-Benzoyl-cAMP, significantly reduced levels of active cdc42, specifically within the periphery of the cell (Fig. 5c–e).

**Figure 6:**
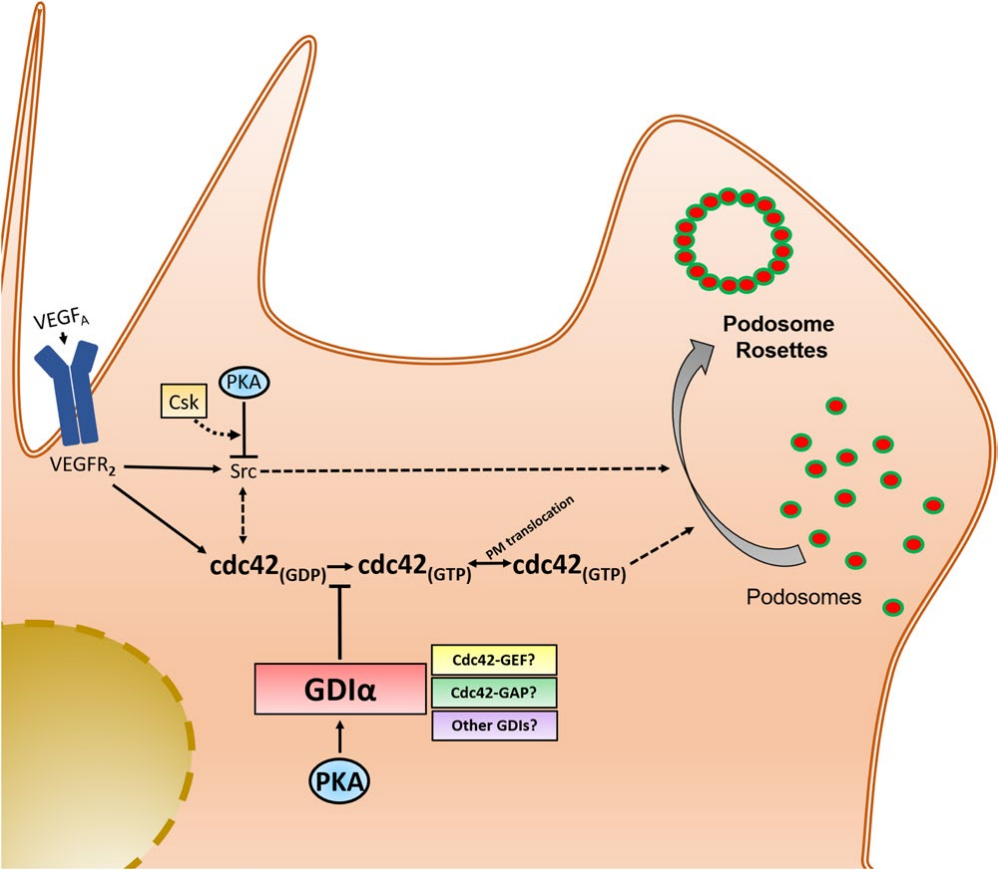
Scheme depicting the novel PKA/GDIα/cdc42 signaling axis and its influence on the invasive EC potential. VEGF binding to its receptor, VEGFR2, results in downstream activation of Src and cdc42. Src activation promotes podosome biogenesis and rosette formation. By promoting GDIα-cdc42 interaction, PKA antagonizes cdc42 activity and restricts the development of podosome rosettes.

Cdc42 cycling between the inactive GDP-bound and active GTP-bound state is largely regulated through its sequestration in the bulk cellular cytosol by virtue of its interactions with Rho-specific guanine nucleotide dissociation inhibitors (RhoGDIs)^52,53^, including the ubiquitously expressed human Rho GDP dissociation inhibitor α (RhoGDIα). Since previous works have identified RhoGDIα as a *bone fide* PKA substrate^54–56^, and phosphorylation of RhoGDIα has been shown to regulate its interaction with RhoGTPases, including cdc42^54–57^, we hypothesized that PKA may reduce cdc42 activity, at least in part, through increased binding of RhoGDIα to cd42. To assess the role of cdc42 interactions with RhoGDIα, we used an alternative FRET biosensor, pTriEx-Antenna- GDI cdc42, which selectively reports cdc42 interactions with the endogenous RhoGDIα^57^. Immunoblot analysis confirmed that treatment of HAECs with N6-Benzoyl cAMP promoted phosphorylation of RhoGDIα at S^174^, and that addition of the PKAi cocktail markedly inhibited this effect (Fig. 5f). Consistent with a role for RhoGDIα in mediating PKA-cdc42 crosstalk, addition of N6-Benzoyl cAMP to cells expressing pTriEx-Antenna- GDI cdc42 increased cdc42 binding to RhoGDIα in the periphery of the cell (Fig. 5g,h), as measured by an increase in the FRET emission ratio. Furthermore, addition of the PKAi cocktail with the PKA-activating cAMP analogue, N6-Benzoyl-cAMP, obviated the increases in cdc42 binding to endogenous RhoGDIα (Fig. 5g,h). Taken together, these data support the novel observation that PKA has an intrinsic negative regulatory role with respect to cdc42 activation, mediated through RhoGDIα, and are consistent with PKA functioning as a cell autonomous “brake” on cdc42-mediated podosome rosette formation and sprouting in ECs (Fig. 6).

## Discussion

In this work, we identify a selective role for PKA in modulating the intrinsic angiogenic sprouting potential of human arterial and microvascular ECs, and in the mouse retinal vasculature. Perhaps more importantly, our findings are consistent with the novel hypothesis that inhibiting baseline levels of PKA activity in these ECs, rather than inhibiting an artificially elevated level of PKA activity following addition of cAMP-elevating agents, was sufficient to dynamically influence their angiogenic potential. We base this interpretation of our data on several findings. First, the influence of knocking down PKA(Cα) on EC podosome rosette formation and angiogenic sprouting was internally consistent with the effects observed when PKA activity was pharmacologically inhibited. In addition, while we report experiments in which N6-Benzoyl-cAMP was used to drive PKA activation and inhibit angiogenic sprouting, no such agent was required to observe the dynamic influence of reduced PKA activity in the human EC cultures. Overall, while our data do not support a critical role for EPAC1 activation or inhibition in regulating the intrinsic sprouting angiogenic activity of these cells, they do identify a potentially modest influence of this cAMP-effector on sprouting. Interestingly, our findings conflict with data presented in two earlier reports that identified a prominent role for EPAC1 in the regulation of angiogenic sprouting^26,58^. While these differences may be attributed to the choice of HUVECs as a model system, or the relatively poor angiogenic responses reported in these earlier studies, further work will be required to formally assess these possibilities.

Although a substantial body of literature details critical roles for both c-Src and cdc42 in numerous angiogenic processes, including those studied here, findings from our work provide novel insight with respect to their relative involvement during angiogenic sprouting in human ECs with varying levels of PKA activity. Overall, our findings confirm previous reports indicating that both Src and cdc42 regulate podosome rosette formation and angiogenic sprouting^59–63^. However, in marked contrast, the regulatory functions of Src and cdc42 varied markedly in ECs with modified PKA signaling. Indeed, in this context, we observed that cdc42 had a dominant role in regulating podosome rosette formation and angiogenic sprouting in cells with reduced levels of baseline PKA activity. Findings in mechanistic studies were inconsistent with the idea that Src was dysregulated in PKA(Cα)-KD HAECs, as its activation by VEGF and sensitivity to inhibition by PP2 were indistinguishable from control-KD cells. Given that a previous report^49^ had convincingly shown that cAMP-elevating agents inhibited Src by promoting PKA-mediated phosphorylating and activation of its inhibitory kinase, namely Csk, our findings were surprising to us. Whether this discrepancy was due to the different ECs used (HAECs and HMVECs *vs* HUVECs), the distinct culture conditions used in the two studies, or differences in the basal activation status of PKA under these distinct conditions, will require further analysis. Rather than minimalizing the functional impact of Src, our findings were more consistent with the idea that knocking down or pharmacologically inhibiting PKA unmasked its role as an active antagonist of cdc42 signaling during podosome rosette formation, matrix remodeling and ultimately EC sprouting angiogenesis. In agreement with this mechanism, activation of HAEC PKA markedly inhibited cdc42 activity in these cells.

During the course of our studies, Nedvetsky and colleagues^36^ reported that EC-specific expression of a cAMP-insensitive (i.e. dominant-negative) PKA regulatory subunit (*Prkar1a-*G324D) inhibited postnatal retinal angiogenesis in the mouse. Though this work reported that the hyper-sprouting phenotype observed in these mice was not related to changes in Notch signaling, they did not establish a molecular basis for their observations. While our strategy to limit PKA catalytic activity in HAECs, HMVECs and in the mouse retinas is distinct from those used in this earlier report, the similarities in our results lead us to speculate that the increased sprouting detected in the *Prkar1a-*G324D mice was likely related to the cdc42-mediated actions that we report here. Future studies investigating the relative impact of selective Src or cdc42 inhibition in the *Prkar1a-*G324D mice may validate our conclusion.

Extensive work has identified a critical role for cdc42 in controlling podosome rosette formation and angiogenic sprouting in ECs; however, it has yet to be determined which of the broad spectrum of cdc42 GEFs, GAPs and GDIs regulate cdc42 function during podosome rosette biogenesis and sprouting. In the context of PKA signaling, we explored the role of RhoGDIα in regulating cdc42 activity, given that it is a known PKA substrate^54–56^, and its phosphorylation status has been shown to regulate RhoGDI interactions with Rho GTPases^54–57^. Consistent with our live cell studies that demonstrate PKA activation reduces cdc42 activity, pharmacologically activating PKA enhanced RhoGDIα binding to cdc42, while PKA inhibition abrogated the RhoGDI-cdc42 interaction. Although these data provide mechanistic insight as to how PKA regulates cdc42 activity, further studies are required to fully characterize the involvement of other GEFs, GAPs and GDIs during podosome rosette biogenesis and angiogenic sprouting. In addition, while our studies demonstrate that a PKA-cdc42 signaling axis plays an important role in the regulation of angiogenic sprouting, it would be essential to explore the role of other GTPases that are likely to influence the angiogenic potential of ECs in the context PKA signaling.

Our work highlights a critical role for the cAMP effector, PKA, in the regulation of angiogenic sprouting. Moreover, since cAMP-signaling is recognized to occur within highly localized cellular domains, this study encourages further work aimed at elucidating the components of the cAMP signalosome involved in regulating this important process. While we chose to investigate the catalytic function of PKA in our studies, previous works have identified a crucial role for the anchoring of PKA-C, via the PKA-RIα subunit, in angiogenic sprouting^36^ and cardiac functioning^64^ in murine models. Further studies will be required to confirm whether PKA-RIα serves as the dominant PKA-R subunit responsible for regulating PKA-C activity in the human ECs used in our *in vitro* models of angiogenesis. In addition, identification of the PKA-R subunit and the associated A-kinase anchoring protein that are critical to help isolate the specific subcellular domain in which the cAMP-signalosome resides to allow for localized GDIα-phosphorylation, and subsequent regulation of cdc42-activity.

In summary, we report that reducing PKA activity in human arterial and human microvascular ECs, and in mouse retinal ECs, increases their angiogenic sprouting potential. We present data inconsistent with the notion that loss of PKA(C) promotes the adoption of a “tip” cell phenotype; rather, our studies support a critical role for enhanced matrix invasion by these cells. At a cellular level, our work identifies increased formation of podosome rosettes in ECs with reduced PKA activity, as well as a commensurate increase in their ability to promote MMP-mediated degradation of the collagen matrix. At a molecular level, we uncouple Src- and cdc42-mediated events in ECs and demonstrate that PKA suppresses sprouting and podosome rosette biogenesis largely by inhibiting cdc42 activities, but not events coordinated by the Src family of kinases. Lastly, we provide mechanistic insight into the regulation of cdc42 activity by PKA. Specifically, we present data that suggests PKA acts to promote sequestration of cdc42 in the cytosol of ECs and that this is coordinated by promoting its interaction with the guanine nucleotide dissociation inhibitor, RhoGDIα. Thus, our work identifies a novel PKA/cdc42-RhoGDIα signaling axis that controls EC angiogenic sprouting, which may represent a novel therapeutic strategy to gain control over angiogenesis.

## Methods

### Cell culture, siRNA transfection

HAECs and HMVECs (Lonza, Walkersville, MD) were maintained in EGM-2 media (Lonza). All cells were cultured at 37 °C in 5% CO_2_. For siRNA transfection, cells were incubated with Lipofectamine 2000 (Invitrogen) and siRNA at a 1:1 ratio per the manufacturer’s instructions. Efficiency of RNAi-dependent knockdown and of RNAi-mediated effects in HAEC or HMVEC were evaluated at either 48 or 72 hours post-transfection, as indicated. The following siRNA targeting constructs were used: PRKACA #1, 5′-GGA AGC UCC CUU CAU ACC AAA GUU U-3′; PRKACA #2 5′-CCA GGU CUU GCU GGU GUAU-3′; PRKACB 5′-GGA AAA AAA CCC UUG GAAC-3′; EPAC1, 5′-AUU GAG AUU CUU CUG CUC CUU GAG G-3′; cdc42 5′-UGA GAU AAC UCA CCA CUGU-3′, as well as a high GC universal negative control (Invitrogen).

### Spheroid angiogenesis assays

HAEC or HMVEC spheroids were generated using a previously established protocol^65^. Briefly, cells were suspended in EGM-2 media containing 0.25% (w/vol) methylcellulose and the experimental compounds, then seeded in a non-adherent round-bottom 96-well plate at a concentration of 1000 cells per well. Once formed, spheroids were harvested and embedded in a collagen gel consisting of 3 vol of collagen stock solution (equilibrated to 2.0 mg/ml) with 1 vol EGM-2 containing 40% FBS, 1 vol 0.25% (w/v) methylcellulose, 25 ng/ml VEGF-A_165_ and experimental compounds. Spheroids were incubated at 37 °C with 5% CO_2_ for 24 hours; live cell images were acquired with a Zeiss Axiovert S100 microscope equipped with a CCD camera and the total number of sprouts per spheroid were manually quantified. Experimental compounds utilized in the assay included a PKA inhibitory cocktail [PKAi cocktail: 200 µmol/L cAMPS-RP triethylammonium salt (ToCris); 1 µmol/L KT5720 (Sigma-Aldrich); 1 µmol/L mPKI (ThermoFisher Scientific); 1 µmol/L H89 (Cedarlane)], N6-Benzoyl cAMP (30 µmol/L; Cayman Chemical), 8-CPT-cAMP (100 µmol/L; Biolog), CE3F4 (20 µmol/L; ToCris), PP2 (10 µmol/L; Millipore), ML141 (10 µmol/L; ToCris), or appropriate vehicles. For mosaic spheroids, HAECs or HMVECs were transfected with the relevant siRNA, labeled with CellTracker™ Green (Molecular Probes), and combined in a 1:1 ratio with control siRNA ECs (pre-labeled with CellTracker™ Red); the mixed-cell suspension was used to generate EC spheroids that were harvested and embedded in collagen, as described above. Live images of mosaic spheroids were acquired after 24 hours. For mosaic spheroid studies, tip cell occupancy by the differentially labeled ECs were quantified for each spheroid, and expressed as a percent of the total number of sprouts generated by the spheroid. In all spheroid experiments, a minimum of 10 spheroids per condition were analyzed for at least three independent experiments.

### RNA isolation and qRT-PCR

HAEC or HMVEC RNA was isolated using a Qiagen RNeasy mini kit; RNA concentration and purity were measured using a NanoDrop 1000 (Thermo Fisher Scientific). cDNA was synthesized with a Qiagen Omniscript RT kit, as per manufacturer’s instructions. For each qPCR reaction, PowerUp™ SYBR™ Green Master Mix (Thermo Fisher Scientific) was used with 10 ng cDNA template and the primers listed in Supplementary Table S1.

### Protein extraction, antibodies and immunoblotting

Proteins were extracted using a triple detergent lysis buffer containing (1% Igepal, 10 mmol/L sodium pyrophosphate, 10 mmol/L sodium β-glycerophosphate, 5 mmol/L benzamidine,10 mM sodium orthovanidate, 50 mmol/L TrisHCl, 150 mmol/L sodium chloride, 0.5% sodium deoxycholate, 0.1% SDS, 10 mmol/L sodium fluoride 0.1 mg/ml PMSF, 1 µg/ml pepstatin A, 1 µg/ml E-64, 5 µg/ml bestatin, 1 µg/ml aprotinin, 1 µg/ml leupeptin); 10–25 µg of extracted cellular proteins were resolved by SDS-PAGE and electro-transferred to polyvinylidene difluoride membranes (Millipore) prior to incubation with antisera. Membranes were blocked with 5% BSA in tris-buffered saline with tween 20 for 1 h prior to an overnight incubation with the primary antibody. The next day, membranes were incubated with a horseradish peroxidase-conjugated secondary antibody (Thermo Fisher Scientific), followed by detection with ECL plus reagents (Perkin Elmer) in accordance with the manufacturer’s protocol. Immunoblots were scanned and densitometry was performed using ImageJ software. Primary antibodies used for immunoblotting are list in Supplementary Table S2.

### Fluorescence resonance energy transfer (FRET)-based analysis of cdc42 activity or interaction with GDIα

FRET-based measurements of cdc42 activity were carried out as follows. HAECs were transiently transfected with the pTriEx4-Cdc42-2G sensor (a gift from Olivier Pertz; Addgene plasmid #68814) or pTriEx-Antenna- GDI cdc42 (a gift from Klaus Hahn; Addgene plasmid #101681) using TransfeX (ATCC) in accordance with manufacturer’s instructions to assess cdc42 activity and cdc42-RhoGDI binding, respectively. Following transfection, cells were plated on glass coverslips coated with type I collagen (20 µg/ml) and imaged at room temperature 24 hr post transfection. Real-time FRET was performed using the Leica DMi8 inverted microscope equipped with a HC PL FLUOTAR 40×/1.30 oil immersion objective, a Leica EL6000 light source and a C11440 ORCA-Flash 4.0 digital camera (Hamamatsu).

Experiments were conducted with cells incubated in Phenol Red-free EGM using the following protocol; resting baseline FRET measurements were captured over 3 minutes, followed by FRET experimental compounds [VEGF (25 ng/ml), N6-Benzoyl-cAMP (30 µmol/L), or PKAi cocktail] for 5–10 min. Three filter sets (CHROMA) were used to acquire images: for CFP, excitation filter 430/424 nm, emission 470 nm; for FRET (CFP/YFP) cube, excitation 430/24 nm, DC 440 nm; 520 nm, emission 540 nm, for YFP, excitation 500/520 nm, emission 535 nm. Images were acquired every 5 seconds with an exposure of 449 ms and processed using LAS X Version 2.0.0.14332 software (Leica). FRET-based measurements were quantified by defining a single region of interest (ROI) per cell (outlining ~10–15um from the outer membrane of the cell). FRET was measured in the selected ROI of each image acquired by capturing fluorescence in three channels; CFP for direct donor excitation and emission, YFP-FRET for donor-sensitized acceptor emission and YFP for direct acceptor excitation and emission. Calculation of the FRET efficiency was determined by performing a background correction in each fluorescence channel captured by subtracting the background fluorescence intensity in a ROI that contained no cells from the emission intensity from the cells expressing the biosensor. FRET emission ratios (YFP-FRET/CFP) were calculated for each time point and normalized over the time course by dividing the emission ratio at each time point by the value preceding drug application. Data is presented as either single representative tracings from individual cells, the mean of averaged tracings from multiple cells in a single experiment, or the area under the curve following drug addition. Mean changes in FRET were obtained by determining the maximal peak increase following drug application from the preceding baseline.

### EC migration and invasion assays

For migration assays, siRNA-transfected HAECs were plated on the upper chamber of a collagen I coated (20 µg/ml) 8 μm porous FluoroBlok™ membrane insert (BD Biosciences). ECs were incubated for 2 hours at 37 °C with 5% CO_2_ to allow for adherence to the upper surface of the membrane. Following cell adhesion, VEGF-A_165_ and FBS (10 ng/ml and 0.5%, respectively) were added to the lower chamber to stimulate migration; after 4 h, the membranes were fixed in 4% paraformaldehyde. For invasion assays, siRNA-transfected HAECs were suspended in type I collagen (2 mg/ml) and this suspension was added to the upper chamber of an 8 μm porous membrane insert; the inserts were incubated at 37 °C with 5% CO_2_ for 1 hour to allow collagen polymerization. Once the collagen had polymerized, inserts were transferred to a 24-well plate in which the lower chamber containing VEGF-A_165_ and FBS (50 ng/ml and 1%, respectively) to stimulate EC invasion of the solidified matrix; after 48 hours the membranes were fixed in 4% paraformaldehyde. In both the migration and the invasion assays, following fixation, HAECs were washed, permeabilized in 0.1% Triton X-100, blocked in 0.3% BSA, then incubated with TRITC-conjugated phalloidin and 4′-6′-diamidino-2-phenylindole (DAPI) to stain actin and nuclei, respectively. The cells which had migrated or invaded to the underside of the porous membrane were imaged with a Zeiss Axiovert S100 microscope and counted. At least three experimental replicates were tested for three independent experiments. To delineate whether treatments altered selectively either migration or invasion, an invasive index was calculated from the migration and invasion data, as described previously^39^.

### Collagen degradation assays

siRNA transfected HAECs, labeled with a live cell nuclear dye (HOESCHT 33342), were trypsinized, resuspended in complete growth medium, and seeded on collagen I-coated coverslips supplemented with DQ-Collagen-I (25 µg/ml). Once the HAECs had adhered to the coverslips (20 min), cells were treated with select experimental compounds [VEGF-A_165_ (25 ng/ml), GM6001 (10 µmol/L; MedChem Express) or appropriate vehicles] for 4 hours. Following treatment, HAECs were briefly fixed in 4% paraformaldehyde (15 min) then imaged using a Zeiss Axiovert S100 microscope; immediate imaging post-fixation optimized visualization of fluorescent products derived from of DQ-collagen degradation. For these studies, collagen degradation was quantified as the average fluorescence intensity per cell for a minimum of 100 cells per experiment using ImageJ software. In some experiments, following imaging for collagen degradation, HAECs were washed, permeabilized in 0.1% triton, blocked in 0.3% BSA and stained with an anti-cortactin antibody (#05–180, clone 4F11; Millipore). Co-localization of the FITC DQ-Col-I degradation products and of anti-cortactin-staining in cortactin-rich circular structures (rosettes) was visualized with a Zeiss Axiovert S100 microscope.

### Promotion of podosome or podosome rosette formation in ECs cultured in 2-dimensions

Following siRNA transfection, HAECs or HMVECs, were trypsinized and seeded on collagen I-coated coverslips in complete growth medium. Adherent cells were treated with VEGF-A_165_ (25 ng/ml) and select experimental compounds [PKAi cocktail, PP2 (10 µmol/L), ML141 (10 µmol/L) or the appropriate vehicle] for a period of 24 hours, then fixed in 4% paraformaldehyde. To visualize actin and cortactin, the fixed cells were and stained with an anti-cortactin antibody (#05–180, clone 4F11; Millipore) and with TRITC-conjugated phalloidin. DAPI was also used to stain cellular nuclei. For high resolution images of podosomes and podosome rosettes, actin/cortactin-stained cells were imaged with a Leica TCS SP8 confocal laser scanning microscope (Leica Microsystems, Concord, ON, CA). For quantification of podosome-positive and podosome rosette-positive HAECs or HMVECs, images were acquired with a Zeiss Axiovert S100 microscope. Individual podosomes were identified by co-localization of F-actin and cortactin staining in a dot-like distribution (0.5–1 µm)^59^ while podosome rosettes were characterized by individual podosomes clustered into a ring or circular-like structure of dimensions (5–10 µm in diameter)^59^. Identification of individual podosomes or podosome rosettes was quantified from at least three separate experiments in which 50–100 cells were examined per experiment.

### Flow cytometry

Following siRNA transfection, HAECs were trypsinized, washed, and incubated in the presence of MMP14 Alexa Fluor® 488-conjugated antibody (0.1 µg per 10^5^ cells; #FAB9181G, R&D systems) or mouse IgG2B Alexa Fluor® 488-conjugated isotype control (0.1 µg per 10^5^ cells; #IC0041G R&D systems) for 40 minutes in fluorescence-activated cell sorting (FACS) buffer containing 2% FBS. Cells were then washed 2Xs with ice-cold FACS buffer, suspended 1% paraformaldehyde and analyzed by flow cytometry on a SH800S Cell Sorter and FlowJo software. The median fluorescence intensity less the isotype control was compared for each data set for two independent experiments.

### *Ex vivo* mouse retina angiogenesis assay

Isolation of fresh mouse retinas was performed as described previously^66^. Following isolation, the retinas were embedded in solution of type I collagen (2 mg/ml) supplemented with the PKAi cocktail, ML141 (10 µmol/L) or with an appropriate vehicle; embedding the retinas in a three-dimensional matrix supports the vasculature and allows for longer treatment windows prior to fixation^38,67^. Collagen-embedded retinas were incubated for 8 hours at 37 °C with 5% CO_2_, then fixed overnight in 4% paraformaldehyde. Following several Hanks-buffered salt solution (HBSS) washes, retinas removed from collagen, blocked and permeabilized in 5% Goat Serum, 0.2% BSA and 0.2% Triton X-100 for 4 h prior to being incubated with an Alexa Fluor 568-conjugated isolectin B4 antibody (1:500, Thermo Fisher Scientific) at 4 °C overnight. Subsequently, retinas were washed and flat mounted in mowiol on microscope glass slides. The isolectin B4-stained retinal vasculature was imaged with a Zeiss Axiovert S100 microscope equipped with a CCD camera. All procedures were approved by the Queen’s University Animal Care Committee in accordance with Canadian Council on Animal Care guidelines.

### Statistical analysis

Statistical analysis was performed using Prism (GraphPad Prism software). For all graphs, data are presented as means ± standard error of the mean (SEM). All data presented in this study were obtained from a minimum of three independent experiments, unless otherwise stated. The Shapiro-Wilk test was used to assess the normality of distribution of investigated parameters. Statistical significance between 2 groups for non-normally distributed data was analyzed by Mann-Whitney U test, and normally distributed data by a Student’s unpaired t-test. Statistical analysis for >2 groups was conducted by Kruskal-Wallis and Dunn’s test for non-normal data, and one-way ANOVA with Tukey’s test for normal data. Values were considered statistically different at p < 0.05.

## Supplementary information

Supplementary Dataset 1

## Data Availability

The authors declare that all data supporting the findings of this study are available within the paper and its supplementary information files.
