# Supplementary material for: A PKA/cdc42 Signaling Axis Restricts Angiogenic Sprouting by Regulating Podosome Rosette Biogenesis and Matrix Remodeling
Source: Sci Rep. 2019 Feb 20;9:2385. doi: 10.1038/s41598-018-37805-y (PMC6382826; doi:10.1038/s41598-018-37805-y)

A PKA/cdc42 Signaling Axis Dynamically Restricts Angiogenic Sprouting by Regulating Podosome Rosette Biogenesis and Matrix Remodeling

MacKeil JL[1], Brzezinska P[1], Burke-Kleinman J[1], Craig AW[2], Nicol CJB[2], Maurice DH[1,2]*

1. Department of Biomedical and Molecular Sciences, Queen's University, Kingston, Ontario, K7L 3N6, Canada

2. Department of Pathology and Molecular Medicine, Queen's University, Kingston, Ontario, K7L 3N6, Canada

Supplementary Figure S1: PKACα and Cβ actively antagonizes the angiogenic sprouting potential of human ECs *in vitro.* (a) mRNA expression demonstrating PKA(Cα), PKA(Cβ) and EPAC1 knockdown efficiency (n=3 ****$p<0.0001$ in Student's unpaired t-test). **(b)** Immunoblot analysis measuring total PKA(C) or EPAC1 levels following PKA(Cα)- or EPAC1-knockdown; n=3 ***$p<0.001$, ****$p<0.0001$ in Student's unpaired t-test. **(c)** Validation of reduced PKA activity in PKA(Cα)-KD HAECs by examining phosphorylation of the *bona fide* PKA substrate, VASP. Following siRNA-mediated knockdown, cells were serum starved for 4hrs, then treated with N6-Benzoyl cAMP (30µmol/L) for 15min (n=3; *$p<0.05$ in one-way ANOVA). **(d-e)** Quantitation of tip cell occupancy normalized to the overall contribution of HAEC sprouts (n=1 biological replicate with minimum of 10 spheroids analyzed per condition) with accompanying knockdown efficiency (representative blots). **(f)** N6-Benzoyl-cAMP promotes phosphorylation of a *bona fide* PKA phosphorylation site on VASP in HAECs, while the PKAi cocktail inhibits this phosphorylation (n=3; **$p<0.01$ in one-way ANOVA). **(g)** Quantification of HAEC sprouting in control- or PKA(Cα)-KD spheroids treated with 100µmol/L 8-CPT, 20µmol/L CE3F4, or the vehicle; values are normalized to the vehicle treated control (n=3; **$p<0.01$, ****$p<0.0001$ in Kruskal-Wallis and Dunn's test). **(h-j)** Silencing HAEC PKA(Cα) does not alter levels of VEGFR2 **(h)** mRNA or **(i)** protein**,** but **(j)** significantly reduces levels of DLL4 mRNA (n=3; **$p<0.01$ in student's unpaired t-test).

Supplementary Figure S2: Activation status of the Akt, Erk1/2, and VEGFR2 signaling systems in response to VEGF. (A) Representative Western Blot of Akt, Erk1/2 and VEGFR2 activation status in control- and PKA(C)-knockdown HAECs under basal conditions and following VEGF stimulation (25ng/ml) (n=2).

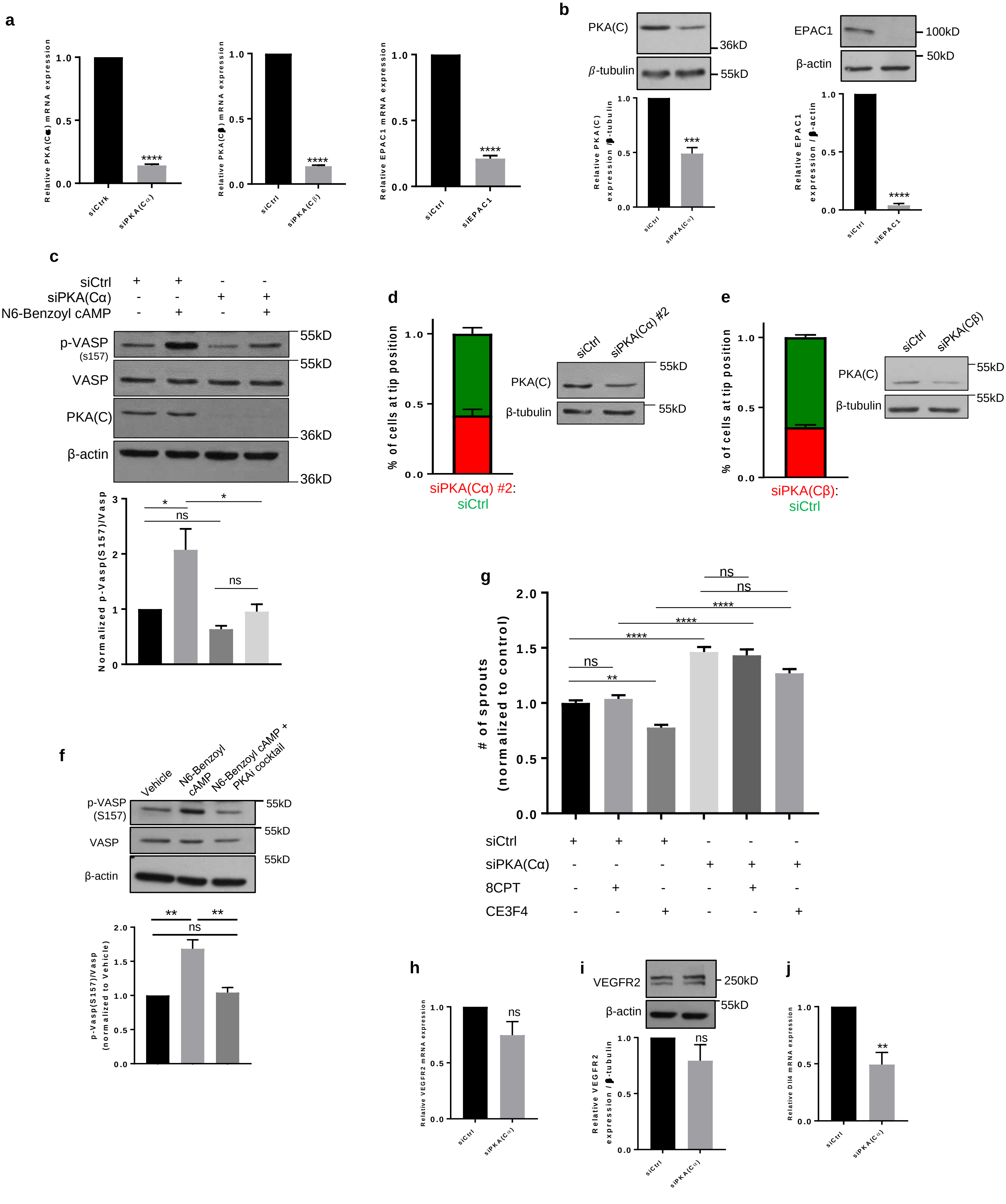

Supplementary Figure S1

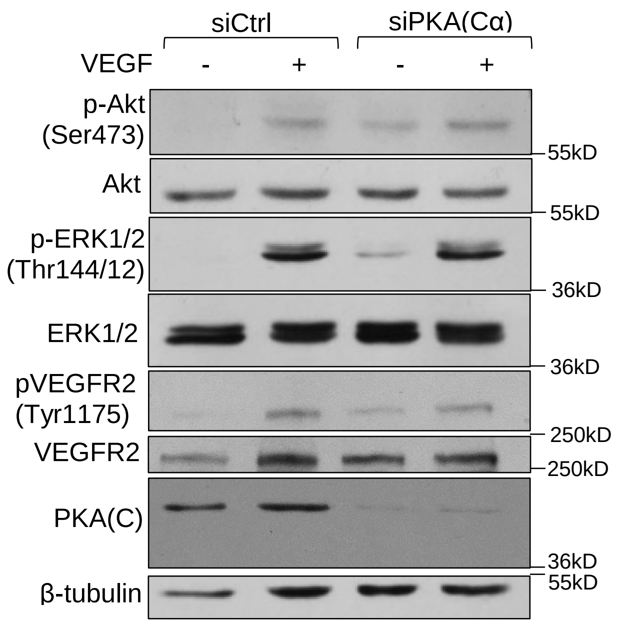

Supplementary Figure S2

Supplementary Table 1: Target genes and primer sequences for qRT-PCR.

Target	Primer set
Dll4	(sense 5′-CTTAGGAGAGAAGGCGCCAC-3′, antisense 5′-GCCTTATACCTCCGTGGCAA-3′
EPAC1	sense 5′-CGACTGGAGCCTCTTCAACA-3′, antisense 5′-CACCCAGTACTGCAGCTCAT-3′
PGK	sense 5′-CTGTGGGGGTATTTGAATGG-3′, antisense 5′-CTTCCAGGAGCTCCAAACTG-3
PRKACα	sense 5′-ATGTTCTCACACCTACGGCG-3′, antisense 5′-CCAGCGAGTGCAGATACTCA-3
PRKACβ	sense 5′-GCAGAACTTGGACATTATGTGG-3′, antisense 5′-CCACCAATCCACTG CTTAT-3′
TBP	sense 5′-TATAATCCCAAGCGGTTTGC-3′, antisense 5′-GCTGGAAAACCCAACTTCTG-3
VEGFR2	sense 5′-CGGTCAACAAAGTCGGGAGA-3′, antisense 5′-CAGTGCACCACAAAGACACG-3′

Supplementary Table 2: Antibodies for immunoblotting.

Target antigen	Vendor or Source	Catalog #	Working concentration
PKAC	BD Biosciences	610980	1µg/ml
EPAC1	Cell Signaling	4155	0.9µg/ml
β-actin	Sigma-Aldrich	A5441	0.3µg/ml
β-tubulin	Sigma-Aldrich	T5293	
VEGFR2	Cell Signaling	2479	26ng/ml
phospho-VASP (S157)	Calbiochem	676604	1µg/mL
VASP	Cell Signaling	3120	0.32ug/ml
MMP14	Abcam	ab78738	1µg/mL
MMP14	R&D Systems	FAB9181G	-
phospho-p44/42 MAP Kinase (Y202/204)	Cell Signaling	9101	0.22ug/ml
p44/42 MAP Kinase	Cell Signaling	9102	11ng/ml
phospho-Akt (S473)	Cell Signaling	9271	77ng/ml
AKT	Cell Signaling	9272	80ng/ml
anti-phospho-Src (Y416)	Cell Signaling	2101	70ng/ml
anti-phospho-Src (Y530)	Santa Cruz	sc-166860	1µg/ml
cSrc	Santa Cruz	sc-8056	1µg/ml
Cdc42	Generous gift from Dr. R. Bhullar, University of Winnipeg	N/A	-
RhoGDIα (phospho-S174)	My Biosource	MBS859500	1µg/mL
RhoGDI	Abcam	AB108977	0.23 µg/mL

Figure 3d

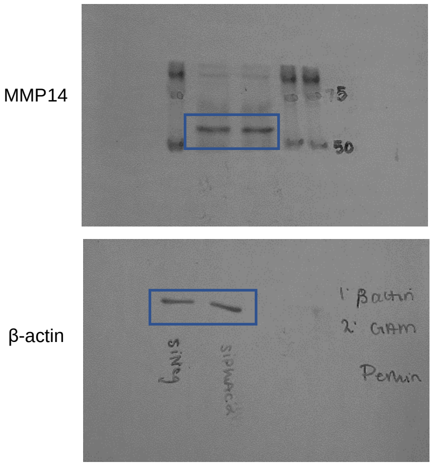

Figure 4f

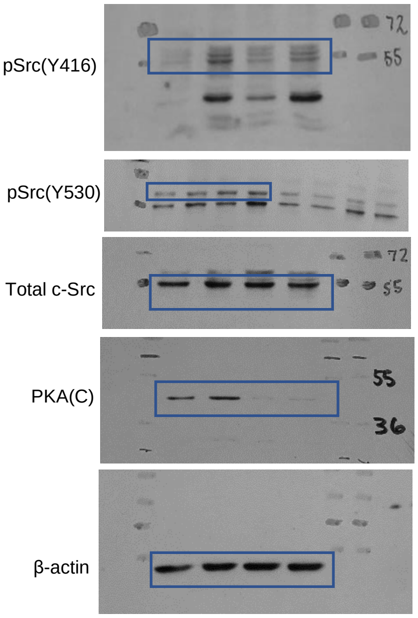

Figure 4i

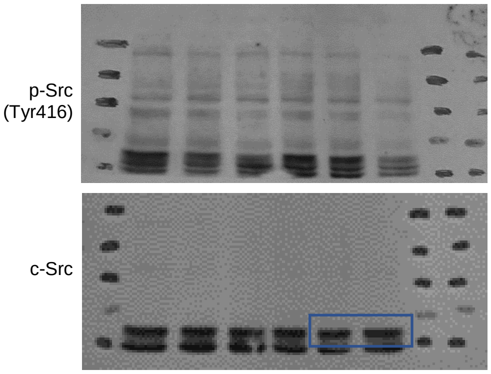

Figure 5f

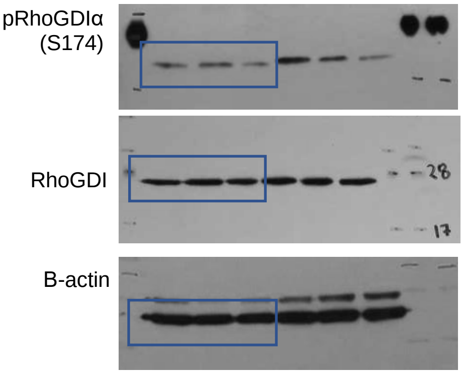

Supplementary Fig. S1b1

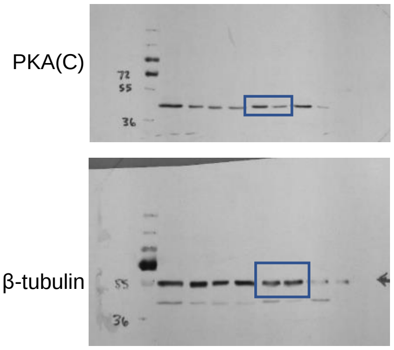

Supplementary Fig. S1b

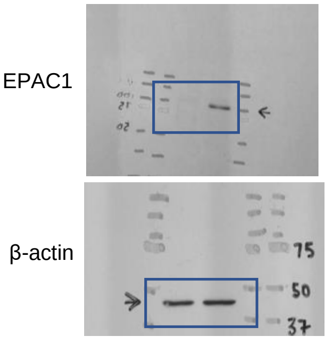

Supplementary Figure S1c

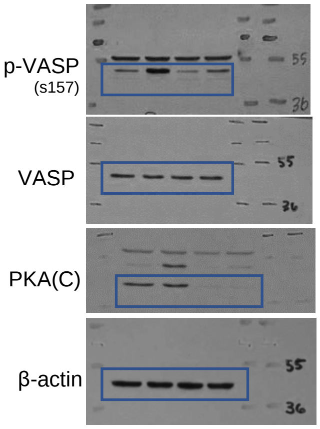

Supplementary Figure S1d

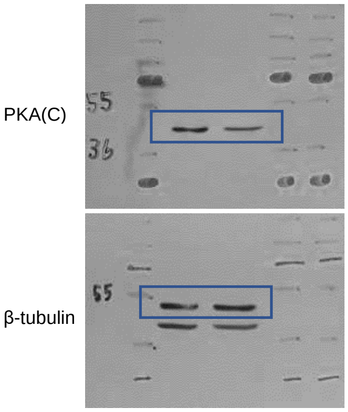

Supplementary Figure S1e

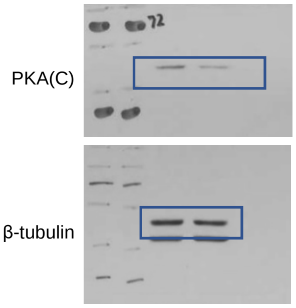

Supplementary Fig. S1f

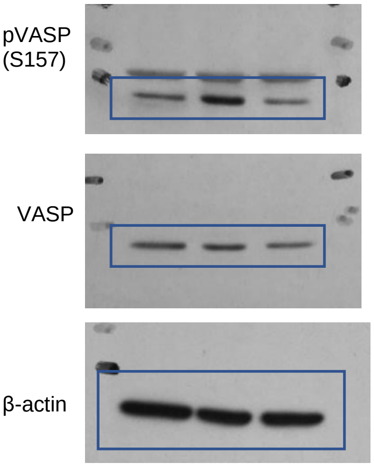

Supplementary Fig. S1i

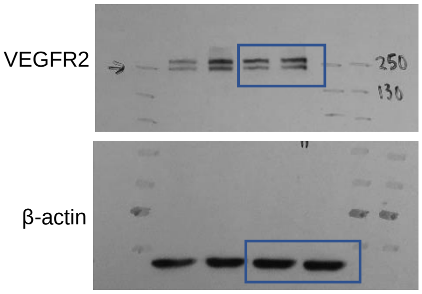

Supplementary Figure S2

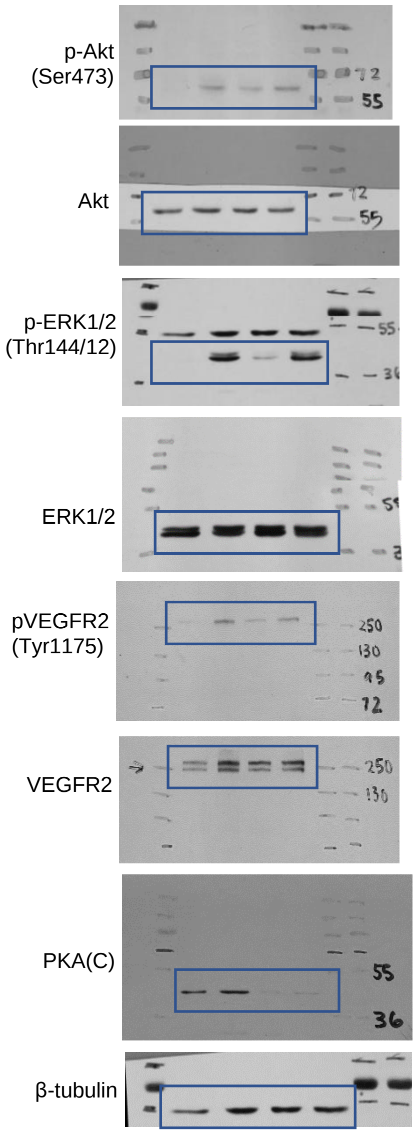

Supplement: Supplementary file 1 — Supplementary Dataset 1 [file 41598_2018_37805_MOESM1_ESM.pdf]
